# Aspectos Atuais da Fibrilação Atrial no Estudo ELSA-Brasil

**DOI:** 10.36660/abc.20240827

**Published:** 2025-02-04

**Authors:** Victor Sarli Issa, Alfredo José Mansur

**Affiliations:** 1 University of Antwerp Antwerp Bélgica University of Antwerp – Cardiology, Antwerp, Flanders – Bélgica; 2 Hospital das Clínicas da Faculdade de Medicina da Universidade de São Paulo Instituto do Coração São Paulo SP Brasil Instituto do Coração do Hospital das Clínicas da Faculdade de Medicina da Universidade de São Paulo, São Paulo, SP – Brasil

**Keywords:** Fibrilação Atrial, Epidemiologia, Estudos Longitudinais

A fibrilação atrial é uma condição clínica associada a manifestações clínicas relevantes, pior prognóstico e aumento de gastos com saúde. A fibrilação atrial não parece ser uma doença homogênea, mas apresenta uma variabilidade significativa em termos de epidemiologia, apresentação clínica, fatores de risco, avaliação do tratamento e prognóstico. Portanto, estudar variações regionais, sociais e étnicas e de alta relevância.^1^

Estima-se que mais de 33 milhões de pessoas em todo o mundo sejam afetadas pela fibrilação atrial^2^ e se espera que a prevalência aumente, já que a população de indivíduos com 65 anos ou mais quase dobrará de 12% em 2010 para uma estimativa de 22% em 2040.^3^ Isso provavelmente trará um ônus adicional para as doenças cardiovasculares e para a fibrilação atrial.^4,5^ Nesse sentido, os dados disponíveis sugerem que a ocorrência da fibrilação atrial no Brasil é pelo menos equivalente à observada em outros países.^6^

É interessante notar que alguns grupos sociais, como afro-americanos e grupos étnicos^7^ originários da Índia, Paquistão, Nepal, Sri Lanka e Bangladesh, que representam mais de 20% da população mundial, parecem ter uma prevalência menor de fibrilação atrial. Possíveis explicações incluem fatores socioeconômicos, menor acesso à assistência médica e determinantes ambientais da saúde; uma base genética foi sugerida, indicando que a menor incidência de fibrilação atrial poderia ser explicada pelo menor tamanho do átrio esquerdo indexado às dimensões corporais^8^ e variações étnicas nos canais iônicos cardíacos.^9^

A apresentação clínica da fibrilação atrial é variável. Atualmente, a fibrilação atrial é categorizada como diagnosticada pela primeira vez (episódios de fibrilação atrial que não foram diagnosticados anteriormente), paroxística (episódios que terminam em 7 dias), persistente (episódios que não terminam em 7 dias) e permanente (episódios para os quais não estão planejadas outras tentativas de restauração do ritmo sinusal). De acordo com diretrizes de sociedades médicas, o diagnóstico de fibrilação atrial exige documentação eletrocardiográfica,^10^ embora a incorporação mais ampla de novas tecnologias, como marca-passos e desfibriladores, *smartwatches* e outros dispositivos de monitoramento, provavelmente exigirá a incorporação de critérios mais flexíveis, à medida que mais informações sobre essas tecnologias se tornarem disponíveis. O uso de diferentes tecnologias e o acesso desigual a elas, por sua vez, imporão desafios adicionais aos médicos, aos pacientes e à sociedade.

O estudo de Boccalon et al.^11^ resulta de uma coorte prospectiva desenvolvida desde 2008 e incluiu 15.105 homens e mulheres, funcionários públicos de universidades ou instituições de pesquisa em seis capitais brasileiras: São Paulo, Belo Horizonte, Rio de Janeiro, Salvador, Rio Grande do Sul e Vitória.^11^ Neste estudo, os autores procuraram identificar dados clínicos, eletrocardiográficos e ecocardiográficos associados à ocorrência de fibrilação atrial.

A coorte representa um estrato relativamente específico, ou seja, funcionários públicos de universidades ou instituições de pesquisa das principais cidades. A análise foi limitada à idade de 74 anos, o que pode ter subestimado a frequência da fibrilação atrial: no presente estudo, a prevalência foi de 4,2%, em comparação com mais de 9% em coortes internacionais de indivíduos com mais de 65 anos de idade.^12^

O diagnóstico de fibrilação atrial foi baseado nos relatos dos pacientes e nos registros eletrocardiográficos na linha de base (2008-2010), bem como nos relatos dos pacientes na reavaliação (2012-2014). Embora isso represente um esforço notável dos autores para identificar casos positivos, a ausência de informações dos prontuários médicos, os métodos eletrocardiográficos contínuos e a sub-representação de pacientes doentes e não ativos podem ter reduzido a taxa de detecção.

No estudo ELSA-Brasil, muitos parâmetros eletrocardiográficos e ecocardiográficos foram associados ao aumento da chance de desenvolver fibrilação atrial no seguimento. É interessante notar que esse não foi o caso de algumas variáveis clínicas e epidemiológicas tipicamente associadas ao aumento do risco de fibrilação atrial, como obesidade e hipertensão arterial. O número relativamente limitado de pacientes diagnosticados com fibrilação atrial (número absoluto de 88 indivíduos) pode ter limitado a identificação de diferenças mais sutis.

Nesse contexto, o presente manuscrito^11^ traz contribuições adicionais e pode ser útil a populações que compartilhem características semelhantes aos participantes desse estudo.
