# Supplementary material for: Aspectos Atuais da Fibrilação Atrial no Estudo ELSA-Brasil
Source: Arq Bras Cardiol. 2025 Feb 4;122(1):e20240827. [Article in Portuguese] doi: 10.36660/abc.20240827 (PMC11809802; doi:10.36660/abc.20240827)
Supplement: Translation

# Translation: Additional Data on Atrial Fibrillation from ELSA-Brasil Study

**DOI:** 10.36660/abc.20240827i

**Authors:** Victor Sarli Issa, Alfredo José Mansur

**Affiliations:** 1 Antwerp Belgium University of Antwerp – Cardiology, Antwerp, Flanders – Belgium; 2 São Paulo SP Brazil Instituto do Coração do Hospital das Clínicas da Faculdade de Medicina da Universidade de São Paulo, São Paulo, SP – Brazil

**Keywords:** Atrial Fibrillation, Epidemiology, Longitudinal Studies

Atrial fibrillation is currently recognized as a medical condition associated with clinically relevant symptoms, worse prognosis, and increased medical costs. Atrial fibrillation does not seem to be a homogenous disease but presents significant variability in terms of epidemiology, clinical presentation, risk factors, access to treatment, and prognosis. Understanding regional, social, and ethnic variations is thus of paramount importance.^1^

It is estimated that more than 33 million individuals worldwide are affected with atrial fibrillation,^2^ and the prevalence is expected to grow as the population of individuals aged 65-year-old or older will almost double from 12% in 2010 to an estimated 22% in 2040.^3^ This will probably bring additional burden for cardiovascular disease and atrial fibrillation.^4^ It has been argued that this health and economic burden might be concentrated in low- and middle-income countries.^5^ In this sense, available data suggests that the atrial fibrillation burden in Brazil is at least equivalent to that seen in other countries.^6^

Interestingly, some social groups such as African Americans and ethnic groups^7^ originating from India, Pakistan, Nepal, Sri Lanka, and Bangladesh - which represent more than 20% of the world's population – seem to have a lower prevalence of atrial fibrillation. Possible explanations comprise socioeconomic, worse access to healthcare, and environmental determinants of health; a genetic basis has been suggested, indicating that lower atrial fibrillation incidence could be explained by smaller left-atrium size indexed to body dimensions^8^ and ethnic variations in cardiac ion channels.^9^

The clinical presentation of atrial fibrillation varies. Currently, atrial fibrillation is categorized as first-diagnosed (episodes of atrial fibrillation that have not been diagnosed before), paroxysmal (episodes that terminate within 7 days), persistent (episodes which are not self-terminating within 7 days), and permanent (episodes for which no further attempts at restoration of sinus rhythm are planned). According to many guidelines, the diagnosis of atrial fibrillation requires electrocardiographic documentation,^10^ even though the widespread incorporation of new technologies, such as pacemakers and defibrillators, smartwatches, and other monitoring devices will probably require the incorporation of more flexible criteria, as more information over these technologies become available. The use of different technologies and the unequal access to them will, in turn, impose additional challenges to physicians, patients, and society.

The study by Boccalon et al.^11^ is the result of a prospective cohort study developed since 2008 and that includes 15,105 men and women, civil servants from universities or research institutions in 6 Brazilian state capitals: Sao Paulo, Belo Horizonte, Rio de Janeiro, Salvador, Rio Grande do Sul, and Vitória.^11^ In the current analysis, the authors sought to identify clinical, electrocardiographic, and echocardiographic data associated with the occurrence of atrial fibrillation.

The cohort represents a relatively specific stratum of the population, namely civil servants from universities or research institutions from major cities. Additionally, the analysis was limited to the age of 74 years, which may have underestimated the frequency of atrial fibrillation. In the present study, the prevalence was 4.2% as compared to more than 9% in international cohorts of individuals above 65 years old.^12^

The diagnosis of atrial fibrillation was based on patients’ reports and electrocardiographic recordings at baseline (2008-2010) as well as patients’ reports at reassessment on follow-up examinations (2012-2014). Even though this represents a remarkable effort of the authors to identify positive cases, the absence of information on medical charts, continuous electrocardiographic methods, and underrepresentation of sick, not working patients might have reduced the rate of detection.

In the ELSA-Brasil study, many electrocardiographic and echocardiographic parameters were associated with an increased chance of developing atrial fibrillation in the follow-up. Interestingly enough, this was not the case with some clinical and epidemiological variables typically associated with increased risk for atrial fibrillation, such as obesity and arterial hypertension. The relatively limited number of patients identified with atrial fibrillation (an absolute number of 88 individuals) may hinder the identification of more subtle differences.

With this context, the manuscript^11^ contributes understanding of atrial fibrillation in this setting and may be useful for populations that share the same characteristics.
